# Supplementary material for: Sphingosine Phosphate Lyase Is Upregulated in Duchenne Muscular Dystrophy, and Its Inhibition Early in Life Attenuates Inflammation and Dystrophy in Mdx Mice
Source: Int J Mol Sci. 2022 Jul 8;23(14):7579. doi: 10.3390/ijms23147579 (PMC9316262; doi:10.3390/ijms23147579)
Supplement: Supplementary file 1 [file ijms-23-07579-s001.zip › ijms-1775388-supplementary.pdf]

de la Garza-Rodea et al.
Sphingosine phosphate lyase is upregulated in Duchenne muscular dystrophy, and its inhibition early in life attenuates inflammation and dystrophy in mdx mice.

Supplemental Material

Supplemental Figure S1. Pathological features of Duchenne muscular dystrophy (DMD) in a representative patient in this study.

Supplemental Figure S2. Immunofluorescence detection of SPL in immune cells in skeletal muscle from mdx mice.

Supplemental Figure S3. Ingenuity pathway analysis: Leukocyte trans-endothelial migration.

Supplemental Figure S4. Ingenuity pathway analysis: ECM-receptor interaction.

Supplemental Figure S5. Ingenuity pathway analysis: Chemokine signaling pathway.

Supplemental Figure S6. Ingenuity pathway analysis: FcγR mediated phagocytosis.

Supplemental Figure S7. Ingenuity pathway analysis: Hematopoietic cell lineage.

Supplemental Figure S8. Ingenuity pathway analysis: Cell adhesion molecules.

Supplemental Figure S9. Ingenuity pathway analysis: B cell receptor signaling pathway.

Supplemental Figure S10. Ingenuity pathway analysis: Natural killer cell mediated cytotoxicity.

Supplemental Figure S11. Ingenuity pathway analysis: Focal adhesion.

Supplemental Figure S12. Ingenuity pathway analysis: Axon guidance.

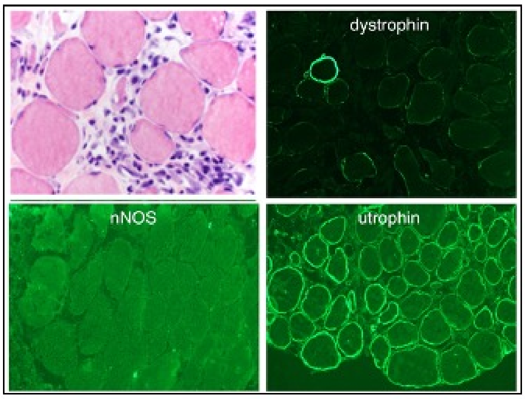

Supplemental Figure S1. Pathological features of Duchenne muscular dystrophy (DMD) in a representative patient in this study. Notable features include inflammatory infiltrates shown by hematoxylin & eosin (H&E) staining (upper left) and immunofluorescence microscopy showing lack of dystrophin expression (upper right; note single revertant fiber), lack of endothelial nitric oxide synthase (eNOS) staining (lower left) and positive utrophin expression (lower right).

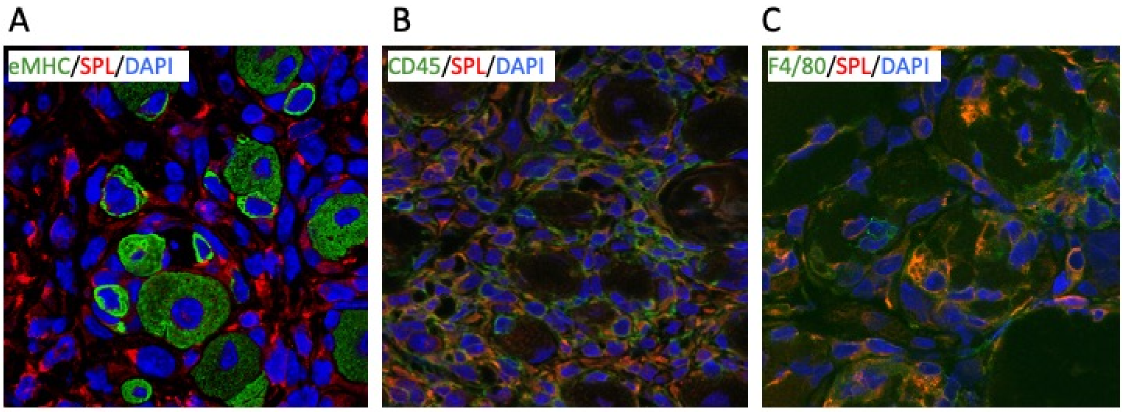

Supplemental Figure S2. Immunofluorescence detection of SPL in immune cells in skeletal muscle from mdx mice. Cryosections of skeletal muscle of four-week-old mdx mice were stained for markers of SPL and either regenerating muscle fibers, infiltrating immune cells, or macrophages. (A) SPL is not found in regenerating myofibers. Green = embryonic myosin heavy chain (eMHC) staining of regenerating myofibers. Red = SPL. Blue = DAPI staining of nuclei. (B) SPL colocalizes with inflammatory infiltrates. Green = CD45 staining of leukocytes. Red = SPL. Blue = DAPI staining of nuclei. (C) SPL colocalizes with macrophages. Green = F4/80 staining of macrophages. Red = SPL. Blue = DAPI staining of nuclei. Magnification = 630X.

25 GENES

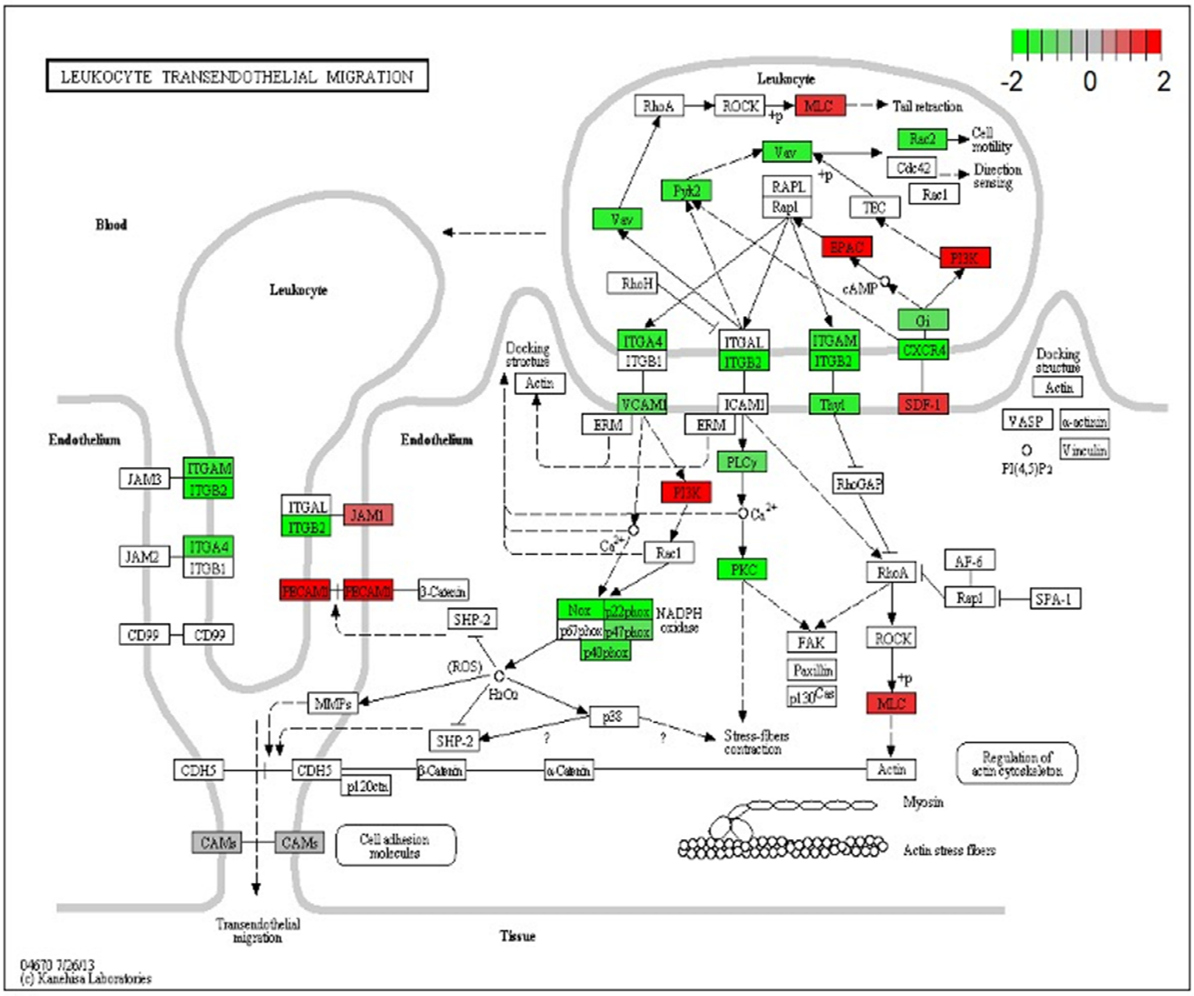

Supplemental Figure S3. Ingenuity pathway analysis of mdx mouse skeletal muscle gene expression changes in response to perinatal LX2931 treatment: Leukocyte trans-endothelial migration.

19 GENES

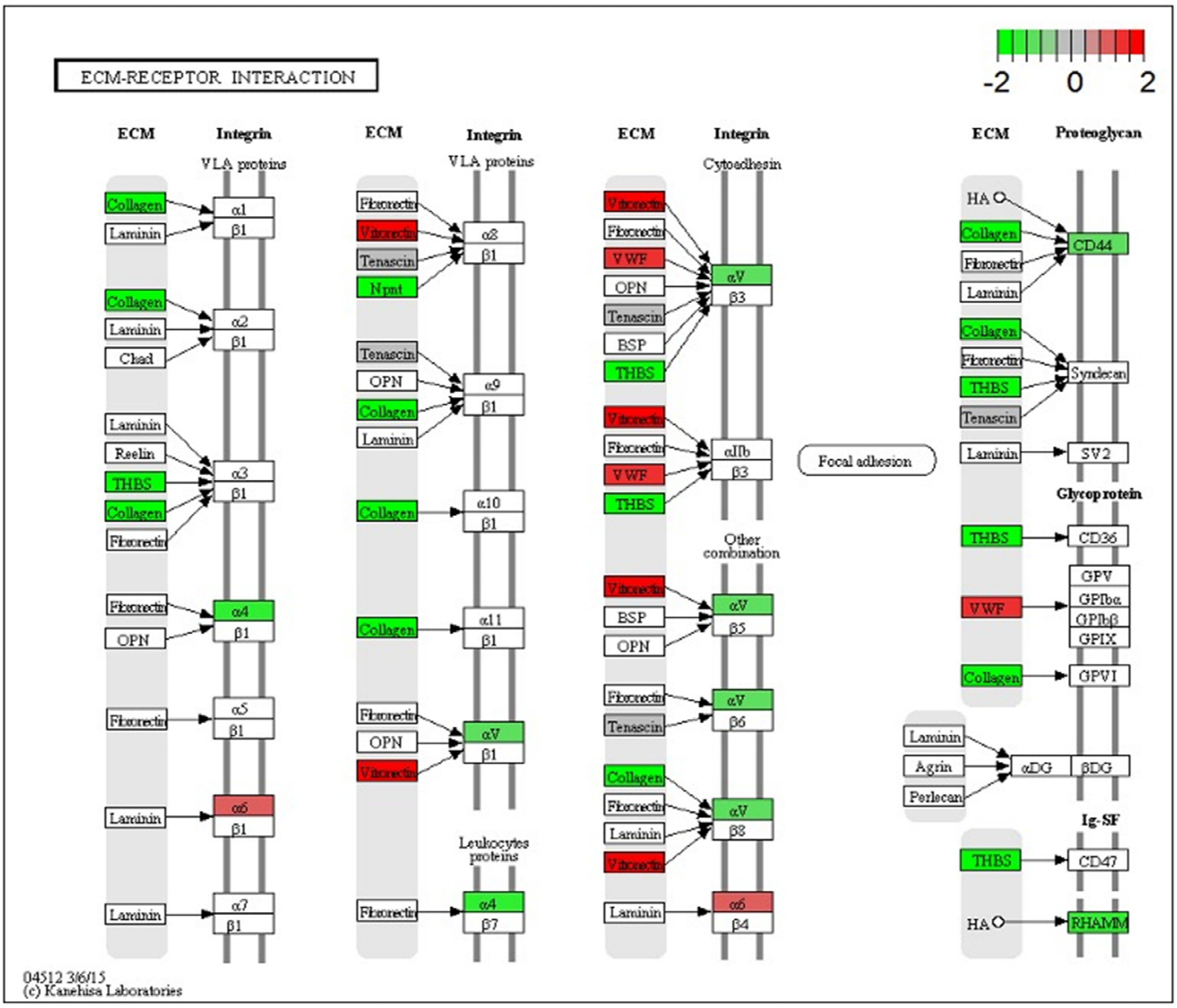

Supplemental Figure S4. Ingenuity pathway analysis of mdx mouse skeletal muscle gene expression changes in response to perinatal LX2931 treatment: ECM-receptor interaction.

29 GENES

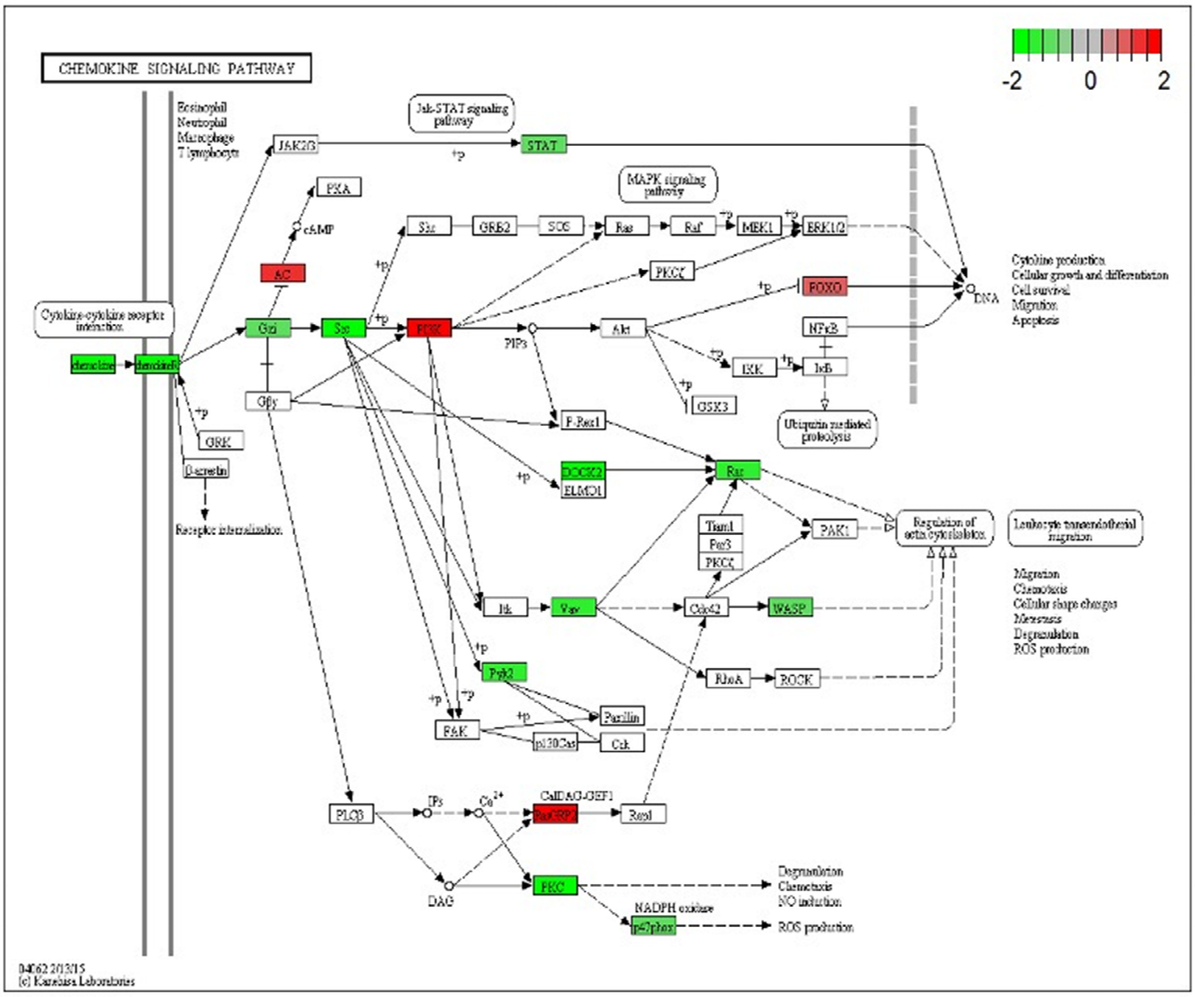

Supplemental Figure S5. Ingenuity pathway analysis of mdx mouse skeletal muscle gene expression changes in response to perinatal LX2931 treatment: Chemokine signaling pathway.

19 GENES

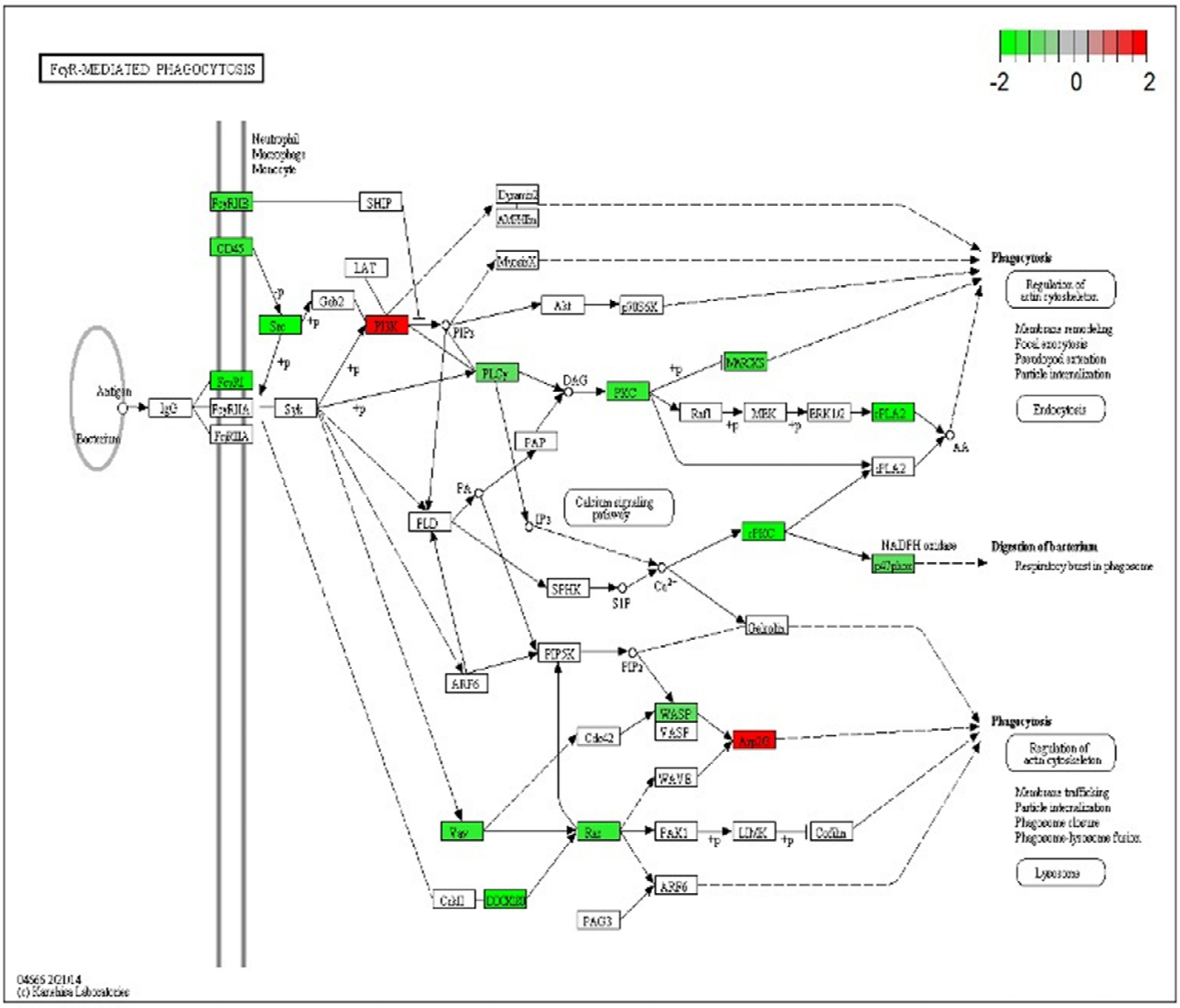

Supplemental Figure S6. Ingenuity pathway analysis of mdx mouse skeletal muscle gene expression changes in response to perinatal LX2931 treatment: FcγR mediated phagocytosis.

17 GENES

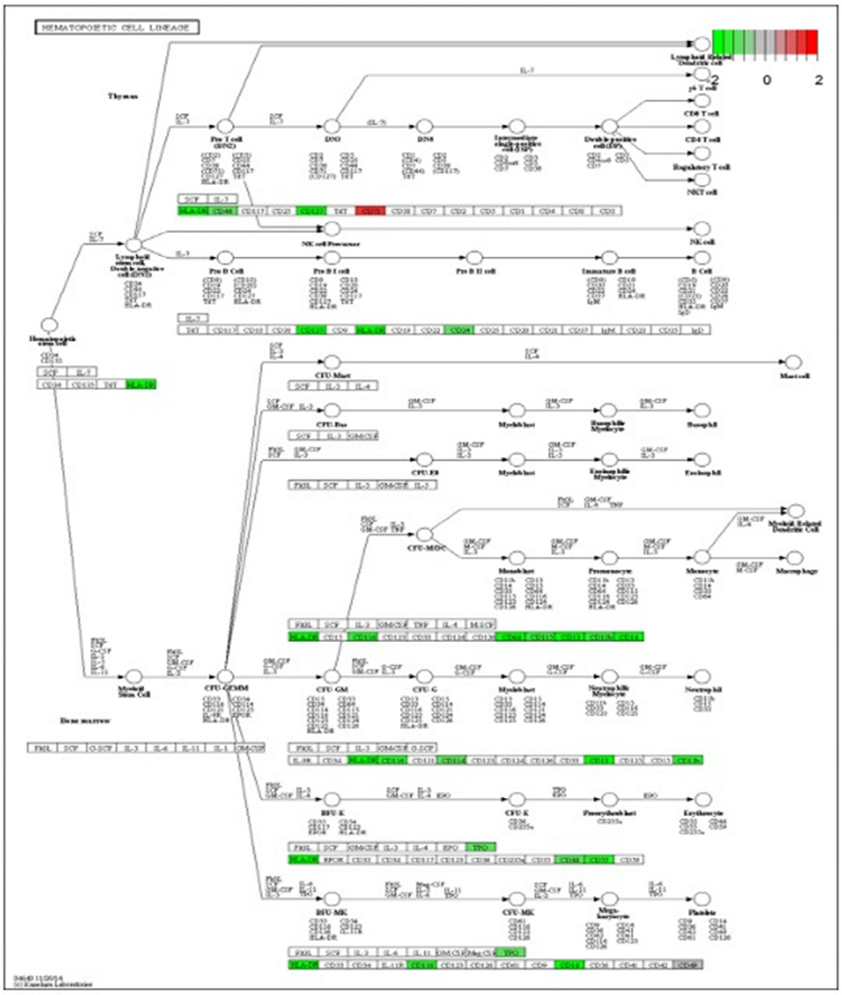

Supplemental Figure S7. Ingenuity pathway analysis of mdx mouse skeletal muscle gene expression changes in response to perinatal LX2931 treatment: Hematopoietic cell lineage.

23 GENES

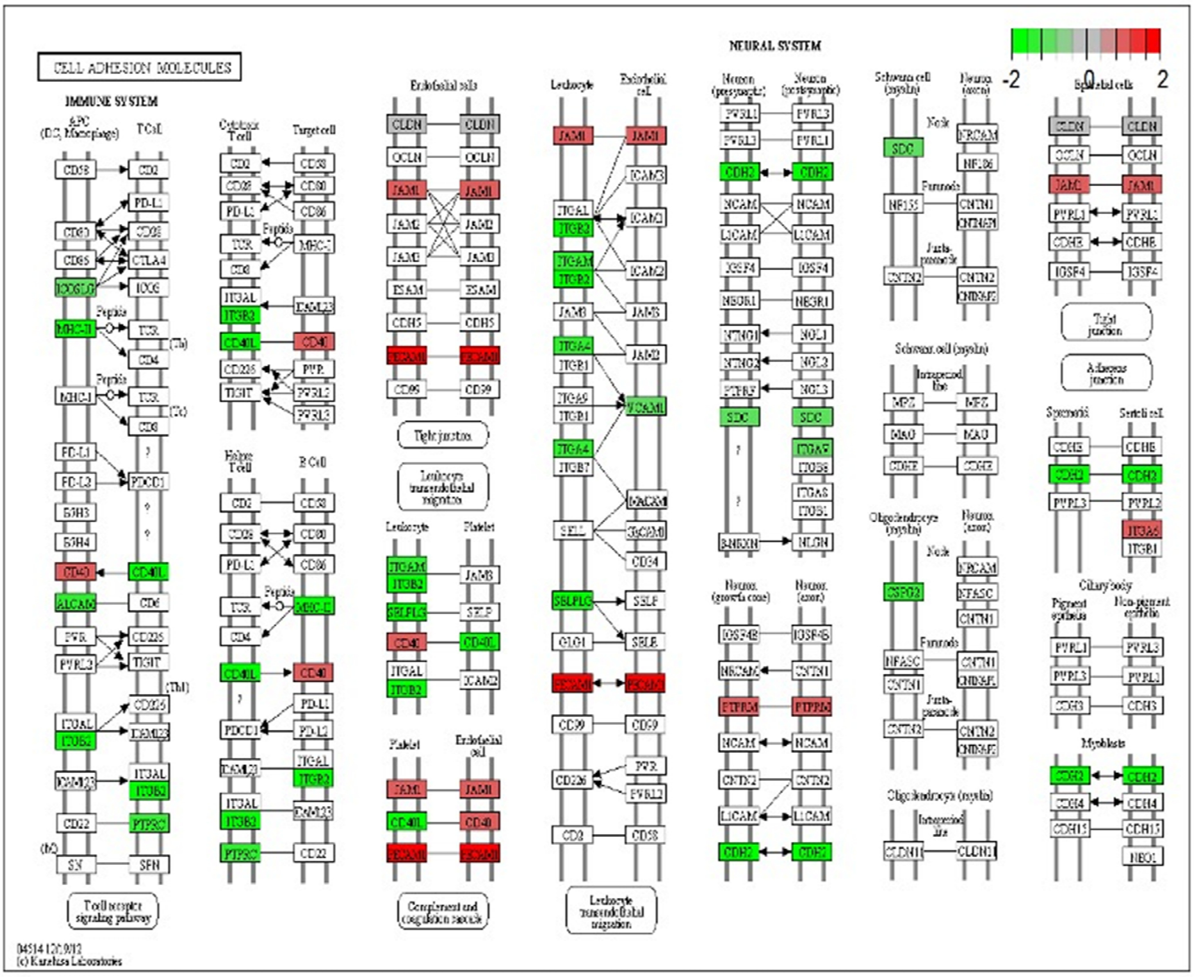

Supplemental Figure S8. Ingenuity pathway analysis of mdx mouse skeletal muscle gene expression changes in response to perinatal LX2931 treatment: Cell adhesion molecules.

15 GENES

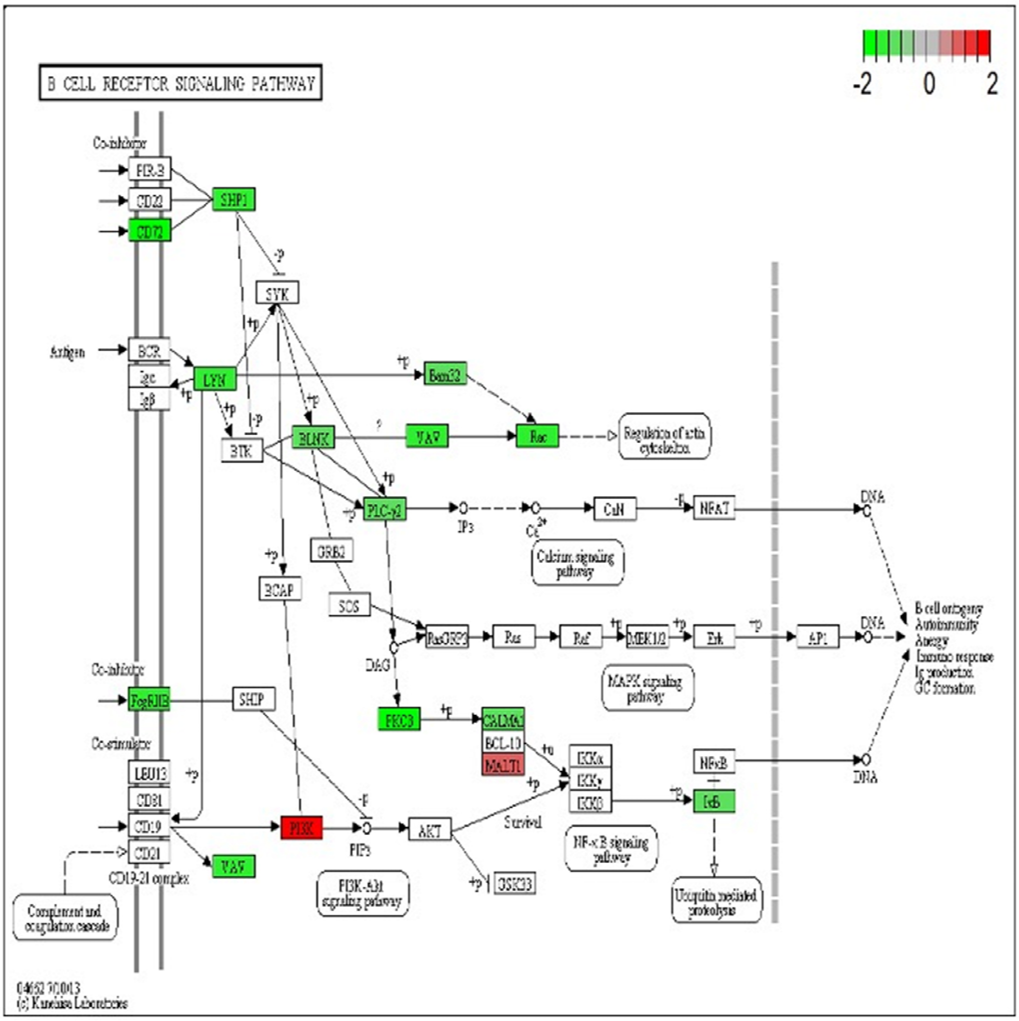

Supplemental Figure S9. Ingenuity pathway analysis of mdx mouse skeletal muscle gene expression changes in response to perinatal LX2931 treatment: B cell receptor signaling pathway.

19 GENES

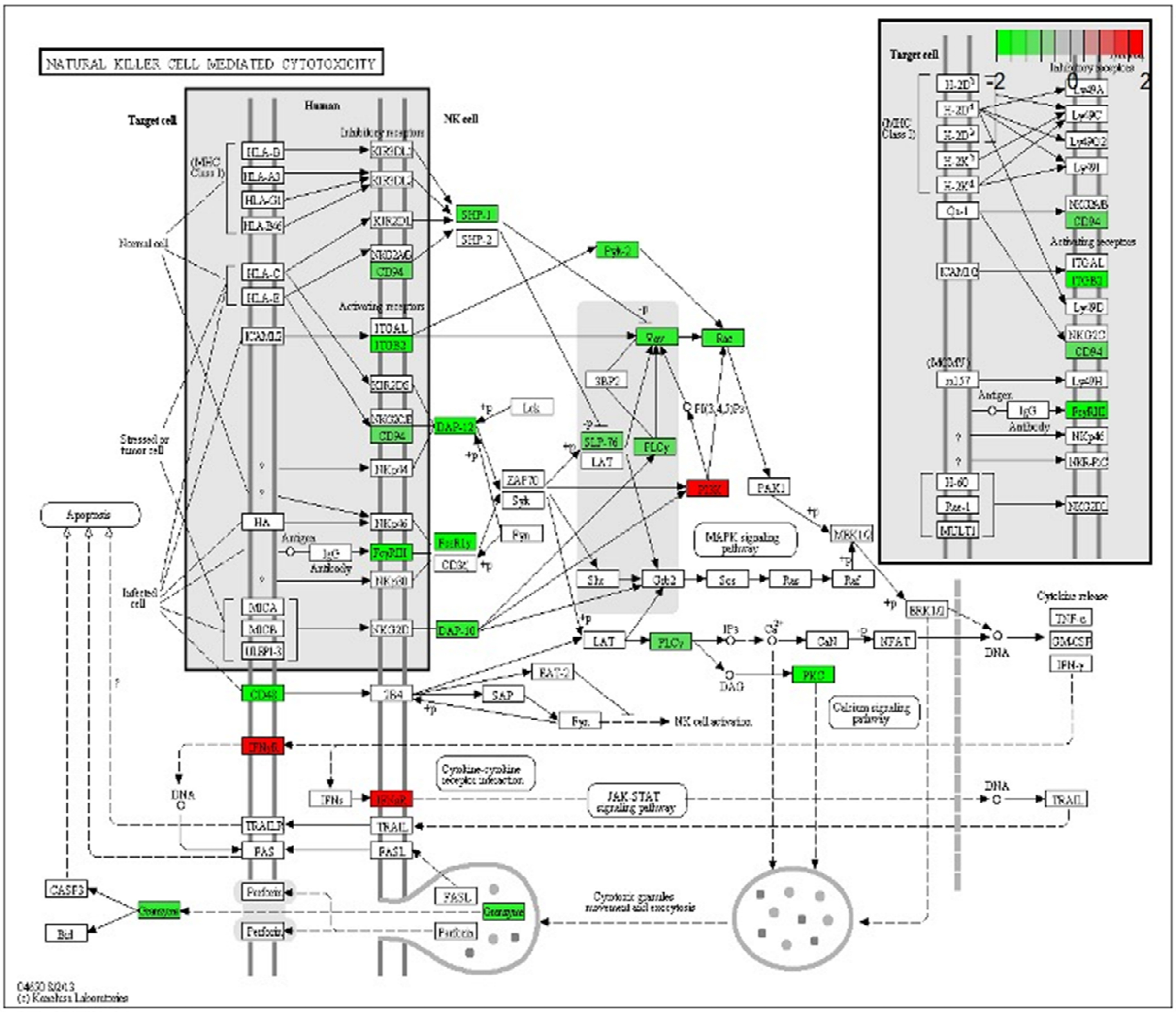

Supplemental Figure S10. Ingenuity pathway analysis of mdx mouse skeletal muscle gene expression changes in response to perinatal LX2931 treatment: Natural killer cell mediated cytotoxicity.

26 GENES

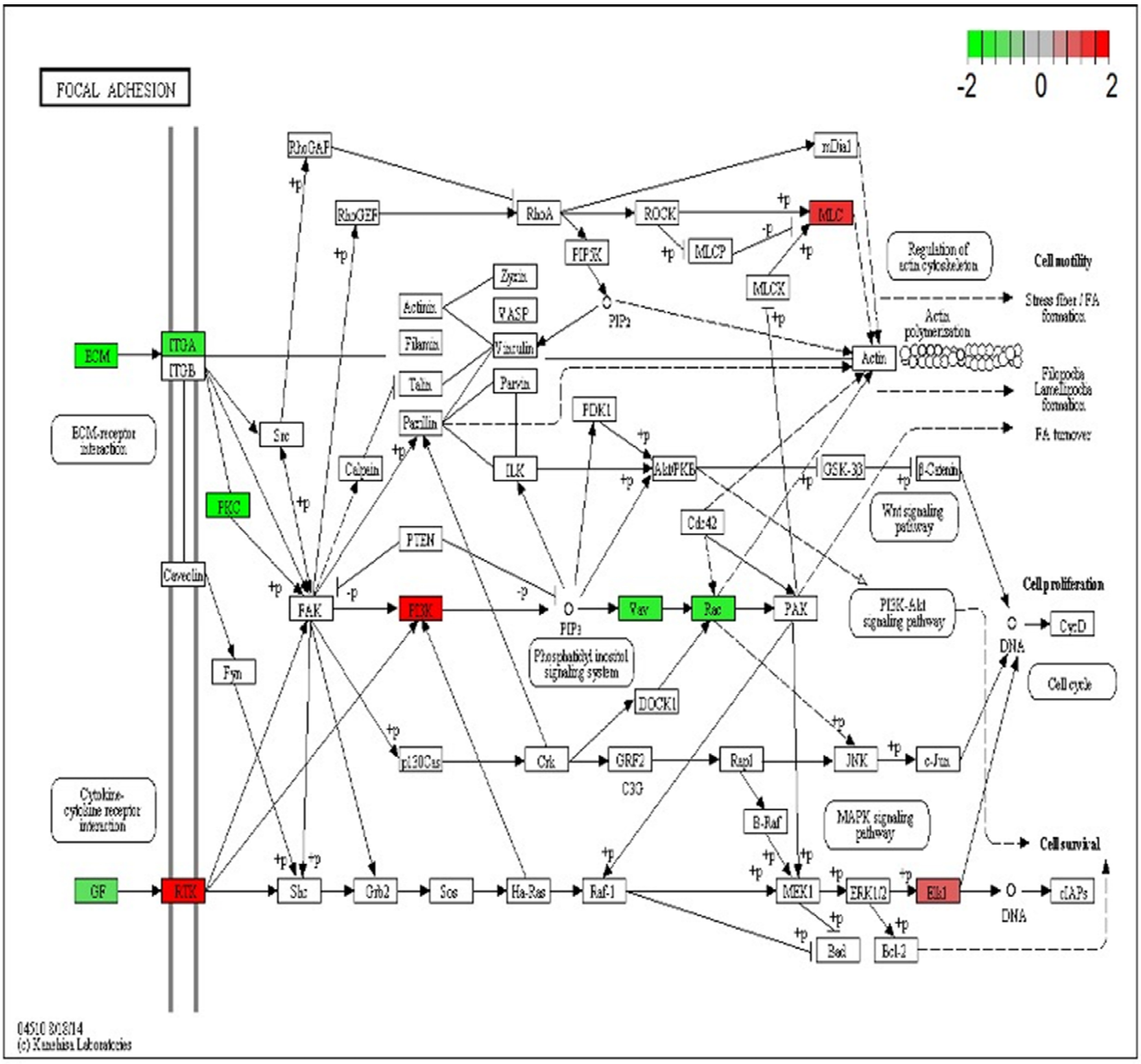

Supplemental Figure S11. Ingenuity pathway analysis of mdx mouse skeletal muscle gene expression changes in response to perinatal LX2931 treatment: Focal adhesion.

19 GENES

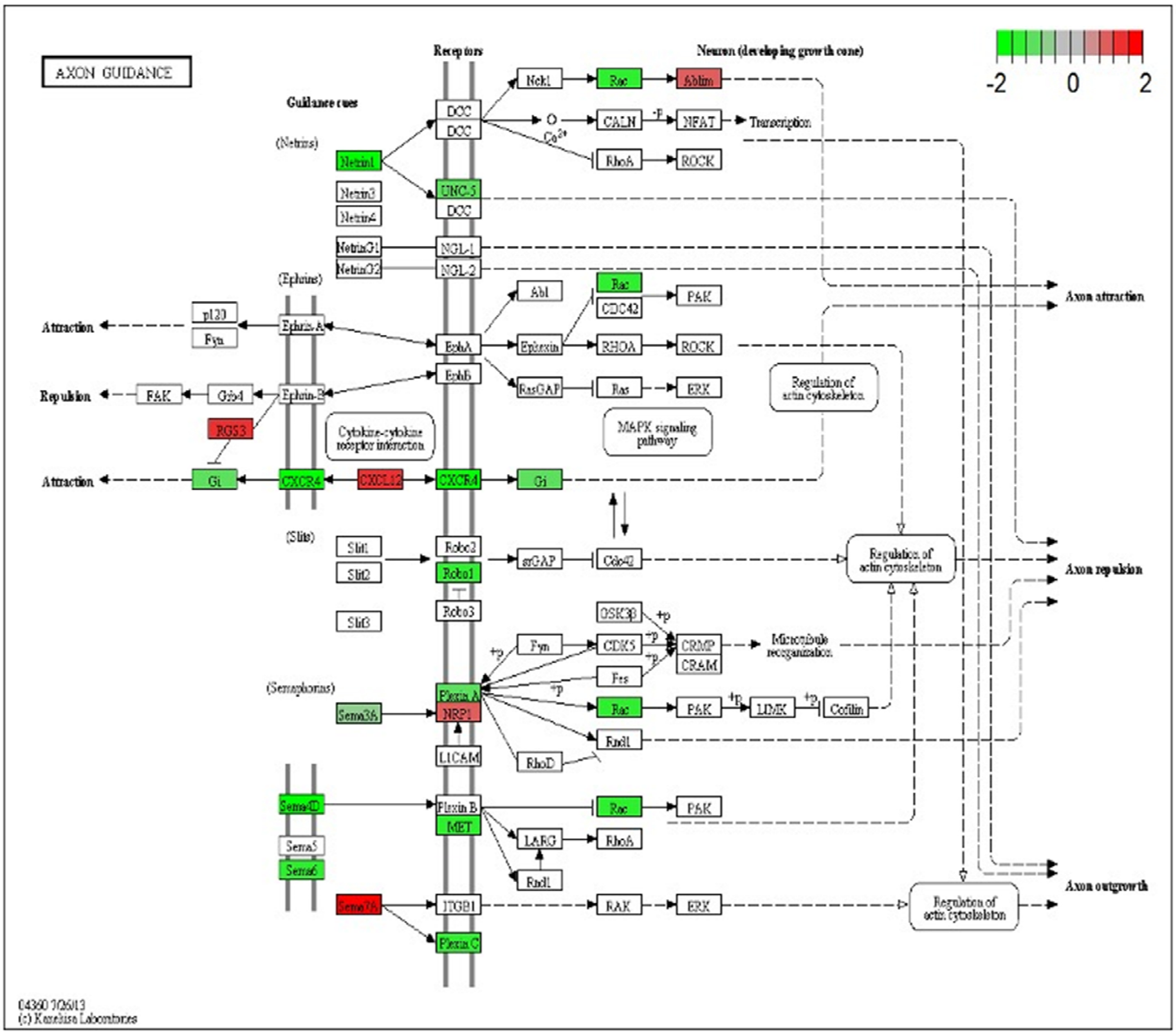

Supplemental Figure S12. Ingenuity pathway analysis of mdx mouse skeletal muscle gene expression changes in response to perinatal LX2931 treatment: Axon guidance.
